# Protein prognostic biomarkers in stage II colorectal cancer: implications for post-operative management

**DOI:** 10.1038/s44276-024-00043-z

**Published:** 2024-02-13

**Authors:** Aziz A. A. Alnakli, Abidali Mohamedali, Benjamin Heng, Charles Chan, Joo-Shik Shin, Michael Solomon, Pierre Chapuis, Gilles J. Guillemin, Mark S. Baker, Seong Beom Ahn

**Affiliations:** 1 Macquarie Medical School, Faculty of Medicine, Health and Human Sciences, Macquarie University, North Ryde, Sydney, NSW Australia; 2 School of Natural Sciences, Faculty of Science and Engineering, Macquarie University, North Ryde, Sydney, NSW Australia; 3 Department of Anatomical Pathology, NSW Health Pathology, Concord Hospital, Sydney, NSW Australia; 4 Concord Institute of Academic Surgery, Concord Clinical School, Faculty of Medicine and Health, Concord Hospital, University of Sydney, Sydney, NSW Australia; 5 Department of Tissue Pathology and Diagnostic Oncology, Royal Prince Alfred Hospital, Camperdown, Sydney, NSW Australia; 6 Department of Colorectal Surgery RPAH & Institute of Academic Surgery at Sydney Medical School, University of Sydney, Sydney, Australia; 7 Institut Pertanian Bogor University, Bogor, Indonesia

## Abstract

Colorectal cancer (CRC) poses a significant threat to many human lives worldwide and survival following resection is predominantly stage dependent. For early-stage cancer, patients are not routinely advised to undergo additional post-operative adjuvant chemotherapy. Acceptable clinical management guidelines are well established for patients in pTNM stages I, III and IV. However, recommendations for managing CRC stage II patients remain controversial and many studies have been conducted to segregate stage II patients into low- and high-risk of recurrence using genomic, transcriptomic and proteomic molecular markers. As proteins provide valuable insights into cellular functions and disease state and have a relatively easy translation to the clinic, this review aims to discuss potential prognostic protein biomarkers proposed for predicting tumour relapse in early-stage II CRC. It is suggested that a panel of markers may be more effective than a single marker and further evaluation is required to translate these into clinical practice.

## Introduction

Colorectal cancer (CRC) is the third most commonly diagnosed cancer and the second leading cause of cancer-related death worldwide, accounting for 9.4% of all deaths from cancer [1]. The classification of tumours according to the extent of tumour spread by clinicopathological staging is the most reliable indicator of prognosis and remains the major determining factor for the management of CRC. The diagnosis and prognosis of patients with stages I, III and IV are well-defined compared to patients with stage II (CRC-II) tumours. CRC-I is routinely managed by surgery alone with approximately a 6% recurrence rate [2], while CRC-III and CRC-IV are considered for post-operative adjuvant and/or targeted chemotherapy. In patients with CRC-II, however, some 20% have been shown to relapse. Most national guidelines do not recommend adjuvant chemotherapy based on risk analysis [3].

**Fig. 1:**
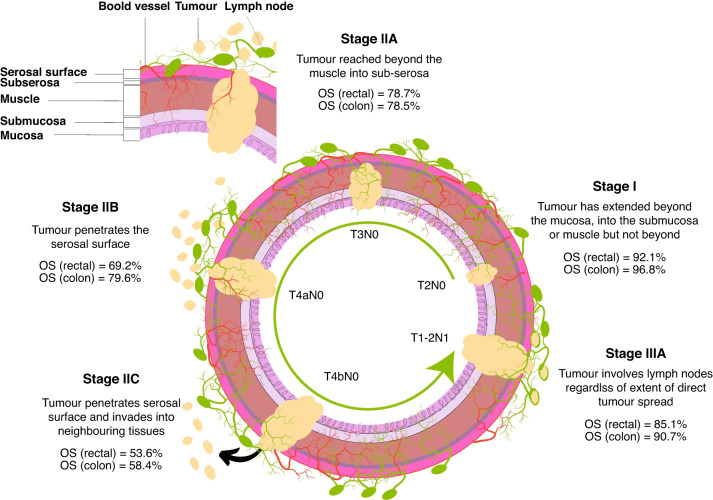
Histopathological features of CRC-II substages according to the TNM staging system. The figure shows CRC-II substages along with corresponding histopathology and relative overall survival (OS) according to each substage as per the 8th edition of the AJCC staging manual [4]. For comparison purposes, stage I and stage IIIA are also included. Histologically, a tumour is still confined to the colorectal wall in the early stages of CRC (CRC-I and CRC-II) while spreading to the adjacent lymph nodes in stage III. In stage I (T2N0), a tumour has only invaded the muscle. In CRC-IIA (T3N0), a tumour extends only to the serosa while penetrating the serosa in stage CRC-IIB (T4aN0). Stage CRC-IIC (T4bN0) is when a tumour reaches the neighbouring tissues. Stage CRC-IIIA (T1/2N1) is when a tumour is found in 1 to 3 lymph nodes. Notably, relative OS in CRC-IIIA is superior (90.7%) to stage CRC-IIC (58.4%) [4].

This classification has been refined to include subdivision within stages to improve risk stratification. The American Joint Committee on Cancer (AJCC-TNM staging system) subdivides CRC-II into three substages [4], CRC-IIA, CRC-IIB and CRC-IIC, according to the extent of tumour spread through the bowel wall. Substage CRC-IIA is characterised by a tumour invading beyond the muscle but not to the serosal surface (T3). Stage CRC-IIB describes a tumour that penetrates the serosal surface but does not involve adjacent tissues (T4a). Finally, CRC is characterised as CRC-IIC if a tumour extends beyond the serosal surface into neighbouring tissues or organs (T4b). All stage II subcategories have no demonstrable lymph node involvement (N0) or distant metastases (M0). Figure 1 illustrates these substages. A description of substages according to other cancer bodies is described in Supplementary Table 1.

## Current prognosis and management of CRC-II based on histopathological staging

Based on data from the AJCC, advancing stage generally correlates with diminished survival rate. Relative survival data for rectum and colon cancer, as described by the AJCC, are based on analysed information from the US Surveillance, Epidemiology, and End Results (SEER) database (Fig. 1). For colon cancer, survival rates are 87.5% in CRC-IIA (T3N0), 79.6% in CRC-IIB (T4aN0) and 58.4% in CRC-IIC (T4bN0) [4]. For rectal cancer, survival rates are 78.7% in CRC-IIA, 69.6% in CRC-IIB and 53.6% in CRC-IIC.

The usual recommendation for stage II patients is resection of the primary tumour with clear margins. However, there are situations where chemotherapy is highly recommended post-resection for those patients considered at high risk of tumour recurrence. Histopathological features are routinely used to predict high-risk stage II CRC patients. However, these features have been shown not to discriminate high-risk stage II CRC reliably and accurately, as demonstrated by several studies [5–8]. One (*n* = 24,847) [6] showed no significant improvement in OS, irrespective of whether histopathological high- and low-risk patients were treated with chemotherapy. Similarly, a second large stage II study (*n* = 58,133) [7] showed OS did not improve if patients had chemotherapy post-surgery. In direct contradiction, other studies (*n* = 153,110) [8] and meta-analyses (*n* = 183,749) [5] demonstrate chemotherapy significantly improves OS in both histopathological low- and high-risk categories. Despite high-risk features ambiguously predicting stage II survival and/or response to therapy, this classical histopathological assessment remains in use and is utilised by many cancer advocacy groups, including ASCO [9] (American Society of Clinical Oncology), ESMO (European Society of Medical Oncology) [10, 11] and NCCN (National Comprehensive Cancer Network) [12] (Table 1).

**Table 1:** Recommendation of major cancer bodies for the management of CRC-II patients post resection of tumour.

The agency	Risk factors	Adjuvant chemotherapy (ACT) Recommendations
**American Society of Clinical Oncology (ASCO)** [9]	T4 tumour	➢Very high risk: • T4 tumour. • Fluoropyrimidine-based ACT is highly recommended. ➢ High risk: ○ If any of the other risk factors. ○ Fluoropyrimidine-based ACT is recommended. ➢ Addition of oxaliplatin to the flouropyrimidine based ACT is not routine. ➢ 3 or 6 months of treatment with capecitabine and oxaliplatin or fluorouracil, leucovorin, and oxaliplatin is recommended.
	Sampling <12 lymph nodes	
	Perineural or lymphatic invasion	
	Poorly or undifferentiated tumour grade	
	Intestinal obstruction	
	Tumour perforation	
	Grade BD3 tumour budding^a^	
	Mismatch repair deficiency	
	Microsatellite instability	
**European Society for Medical Oncology (ESMO)** [10]	T4 (very high)	➢ Very high risk: • If they are MMS and T4 combined. • If they have more than one risk factors. • In this case, addition of oxaliplatin to the treatment, consider 3 months of CAPOX. ➢ High risk: • T4, number of examined lymph nodes <12, tumour perforation or occlusion, tumour grade 3, or loss of MSI. • Fluoropyrimidine ACT is recommended [104].
	MSS (very high)	
	Number of examined lymph nodes <12	
	Primary tumour perforation	
	Primary tumour occlusion	
	Grade BD3 tumour budding	
	No MSI	
**National Comprehensive Cancer Network (NCCN)** [12]	MSI-H/dMMR	➢ Observation and no ACT.
	MSS/pMMR	➢ Observation and ACT.

^a^Tumour budding (BD) refers to single cells or clusters at the invasive front of colorectal tumour. Grade differs according to size of cluster or cells.

*ACT* Adjuvant chemotherapy, *BD3* Tumour budding grade 3, *dMMR* Mismatch repair deficient, *MSI* Microsatellite instability, *MSI-H* High microsatellite instability, *MSS* Microsatellite stable, *pMMR* Mismatch repair proficient, *T4* Tumour size 4.

For CRC-II substages, a study by Yang et al. investigated the benefit to 116 CRC-II patients who received adjuvant chemotherapy (95% stage IIA) [13] and showed that patients had no significant improvement in OS nor disease-free survival (DFS) (40.33 vs. 40.02 months [*p* = 0.143]) and DFS (38.52 vs. 34.42 months [*p* = 0.187]). Similarly, a study performed primarily on CRC-IIB and IIC patients (92%) showed no significant difference in OS or DFS after additional chemotherapy (DFS, *p* = 0.124; OS, *p* = 0.225) [14].

Interestingly, stage IIC has a worse prognosis than stage IIIA. This histopathological differentiation of CRC tumour appears inconsistent with the corresponding survival data. That is, stage IIC patients have worse relative survival in both rectal and colon cancer than stage IIIA patients (Rectal: 85.1% in CRC-IIIA *vs*. 53.6% CRC-IIC, Colon: 90.7% in CRC-IIIA *vs*. 58.4% CRC-IIC) (Fig. 1). Similarly, multiple studies have also revealed the inconsistency in using pathological staging to evaluate overall survival. For example, stage IIA patients have worse cause-specific survival (CSS) in rectal cancer compared to stage IIIA patients [15]. This study based on an analysis of SEER data included 16,788 patients (13,551 staged IIA and 3,237 staged IIIA) demonstrated that CRC-II patients have a worse CSS than Stage CRC-III (HR 0.894, 95% CI 0.816–0.979, *p* = 0.016). This was particularly evident in the subgroup with fewer than 12 lymph nodes harvested (HR 0.805, 95% CI 0.719–0.901, *p* < 0.001). Moreover, CRC-IIA and CRC-IIC demonstrate significantly higher recurrence risk rates than stage IIIA [16]. Furthermore, CRC-IIA patients are thought to benefit more from chemotherapy than CRC-IIB patients (OS = 84.7% for IIA vs. 72.2% for IIB) [17]. Also, CRC-IIA patients have been observed to have a higher survival rate than CRC-IIB and CRC-IIC (OS = 84.7% for IIA *vs*. 72.2% for IIB) [18].

Given these inconsistent results, different cancer organisations worldwide have proposed variable recommendations for treating CRC-II patients. For the ASCO and ESMO, tumour size pT4 is mutually used to indicate the group with the highest RR and including a minimal harvest of 12 uninvolved nodes in the resection specimen. Perforation and obstruction of the intestine are also two other factors that are considered (Table 1). Both the ASCO [9] and the ESMO [10] consider T4 tumours to be involved in assigning the very high-risk CRC-II group. ESMO strongly recommends chemotherapy for CRC-II patients if any T4 tumour is accompanied by high Microsatellite stability (MMS). Both recommend chemotherapy if any other risk factors are detected in the resected tumour.

Mismatch repair (MMR) genes are responsible for detecting and correcting mismatches during replication and if mutated results in high microsatellite instability (MSI) [19]. Most cancer organisations recommend patients with high MSI to undergo chemotherapy after tumour resection to reduce RR. For this reason, MSI is considered as a molecular risk factor; indeed, it is the only way in which the National Comprehensive Cancer Network (NCCN) recommends distinguishing between low- and high-risk groups of CRC-II patients.

Overall, there remains a need for standardised and consistent guidelines that delineate the high-risk group (whose tumours recur after resection). This delineation is essential as the evidence that establishes the criteria for delineation remains contradictory in the literature. These contradictions may be attributed to differences in the type of guidelines followed or the high cost resulting in insufficient available information.

These discrepancies emphasise an unmet clinical need to identify molecular markers that distinguish CRC stages II and III more precisely. Furthermore, most patients whose tumours recur are less likely to be cured as the tumour is usually detected late and in a more aggressive form [20]. It has been suggested by Parent et al. and others [21–23] that some form of molecular markers are required to accurately distinguish between high and low-risk of recurrence stage II CRC patients as such markers are more informative than histological observations [21].

Protein markers hold the most potential as they are more easily actionable and routinely used in diagnostic settings. This review aims to investigate how protein biomarkers could discriminate heterogeneity in CRC-II and discusses the limitations of the studies that have precluded their widespread use in the clinic.

## Molecular markers

Several studies have investigated genetic markers for potential use as prognostic markers for CRC-II patients after resection. It is estimated that CRC-II patients who are plasma ctDNA-positive after surgery have an extremely high risk of tumours recurring (HR, 18; 95% CI, 7.9 to 40; *p* = 2.6 × 10−12) if not treated with chemotherapy [24–26]. A recent study showed the benefit of a ctDNA-guided approach in reducing the need for adjuvant chemotherapy for CRC-II patients [27]. Similarly, the status of gene mutations, such as in *KRAS* and *BRAF* genes, have also been studied as indicators for recurrence after surgery. Although *KRAS* mutations showed no significant positive outcomes in 1564 CRC-II and CRC-III patients in a study by Roth et al., (stage corrected HR, 1.05; 95% CI, 0.85 to 1.28; *p* = 0.66) [28], its mutation was shown to be associated with poor prognosis in 511 tumours (HR, 0·81; 95% CI, 0·68 to 0·96; *p* = 0·014) in another study [29]. Such contradictions in results are also demonstrated in the case of *HER2* mutations as a prognostic marker. Feng et al. [30], showed that *HER2* overexpression in 206 patients was associated with better OS and risk-free survival (RFS) in those who received chemotherapy *vs*. those who did not, while in another retrospective analysis of 1914 CRC-II samples found that *HER2* expression was not associated with OS and recurrence [31].

In addition to the genetic markers, some microRNAs (miRNAs) have also been proposed as potential predictive markers for recurrence in CRC-II. It has been shown that miRNA can serve as a reliable marker due to its stability which renders it detectable in the plasma or the tumour. A few studies have demonstrated that high levels of miR-181 [23], miR-21, miR-498 and miR-320 [32] are associated with higher RR and poor OS of CRC-II. Although significant effort has been put into uncovering genetic- or RNA-based markers for recurrence in CRC-II, the premise and ease of translation to the clinic of protein markers has meant that numerous studies have attempted to uncover reliable protein markers that could discriminate patients at high risk of recurrence.

## Protein Candidates

Protein biomarkers are recognised for their seamless integration into pathology labs, surpassing genetic markers in practicality and affordability. Most pathology labs are equipped with Immunohistochemistry (IHC) testing equipment which only requires the development of an antibody with high specificity to a relevant, sensitive and specific protein biomarker. However, the discovery process to uncover new protein biomarkers is arduous.

The biomarker discovery workflow in CRC-II can be divided into 2 parts, candidate selection and subsequent validation. Candidate selection can be made via informatics approaches [33, 34], DNA/RNA microarray data analyses [35], protein involvement in key biochemical processes [36], being differentially expressed through mass spectrometry (MS) analysis [37], Olink proteomics assays [38] or SomaLogic assays [39] or as a diagnostic or prognostic marker for other tumour types [34].

Once the protein has been assigned as a candidate upon the application of stringent criteria [40], technologies such as sequential window acquisition of all theoretical fragment ion spectra (SWATH MS), multiple reaction monitoring (MRM), IHC and ELISA allow the validation of the proposed candidates [41]. In the following section, potential prognostic protein biomarkers proposed to identify low-risk CRC-II patients are considered with the corresponding discovery and/or validation methods, and a summary of the major findings of the studies on candidates published in the literature alongside contradictory ones, if any, are described. Thus far, 10 markers have been proposed in the literature to discriminate high- and low-risk CRC-II (Table 2).

**Table 2:** Protein candidates proposed as potential prognostic markers for high and low risk of tumour recurrence in CRC-II patients.

Candidate	Method used	Sample type	Stage/System	Stats	Study outcome	Ref.
**CDX2**	Bioinformatic/IHC	FFPE Tissue	II/TNM (CC)	DFS (51% *vs*. 80%, *p* = 0.004 for CDX2-negative *vs*. CDX2-positive in the validation cohort). Improvement shown in CDX2-negative groups with and without ACT, *N* = 121, at 5-year follow up	Absence of CDX2 is an indicative of poor prognosis of CRC-II	[33]
	IHC	FFPE Tissue	II/TNM (CRC)	DFS in two cohorts (*p* = 0.0267 and 0.0118), *N* = 1157, at 5-year follow up	Absence of CDX2 is significantly associated with poor prognosis of CRC-II	[48]
	Transcriptome arrays	Fresh-frozen primary tumour	II & III/TNM (CC)	OS: HR 2.02; 95% CI (1.27-3.23); *p* = 0.003; RFS: HR 1.73;95% CI (1.06-2.82); *p* = 0.027, N(discovery)= 469 in discovery N(validation)= 90 at 6-year Follow up	Absence of CDX2 indicates poor prognosis for CRC-II only in CMS4 subtype.	[49]
	IHC	FFPE tissue	II/TNM (CRC)	DFS (*p* = 0.03) *N* = 227, at 5-year follow up	**Contradictory:** High expression of CDX2 is indicative of poor prognosis only when MUC2 is co-expressed	[50]
	Exon-level microarrays analysis/IHC	TMA	II/TNM (CRC)	RFS^a^ (*p* = 0.91), **non-significant** *N* = 422, at 5-year follow up	**Contradictory:** Absence of CDX2 is **NOT** an adverse prognostic biomarker for CRC-II patients	[51]
**CEA**	Meta-analysis	Serum	I/II/TNM (CC)	OS (79.7% *vs*. 64.5%), *N* = 45,449, at 3-year follow up	Preoperative CEA levels ≥ 2.35 ng/mL is associated with poor prognosis for CRC-II patients	[55]
	Meta-analysis	Serum	II/TNM (CC)	DFS (88.5% in the ≤ 2.35 ng/mL and 78.7% in the > 2.35 ng/mL group (*p* = 0.006)) *N* = 899, at a 3-year follow up	Postoperative CEA levels > 2.35 ng/mL is a strong marker for poor prognosis of CRC-II patient	[56]
	Meta-analysis	Serum	II/TNM (CRC)	OS (53.62% *vs*. 84.16%), DFS (50.03% *vs*. 86.75%), and CSS (61.77% *vs*. 90.30%) at 3.66 ng/ml cutoff *N* = 1081 at 5-year follow up	Early postoperative CEA levels of > 3.66 ng/ml is significantly associated with poor prognosis	[57]
**CXCR3**	rtPCR/IHC	FFPE tissue	II/TNM (CRC)	OS (*p* < 0.001), DFS (*p* < 0.0001), *N* = 143, at 5-year follow up	Higher CXCR3 levels is significantly associated with recurrence and poor survival in recurrent CRC-II patients	[60]
	IHC	FFPE tissue	I, IIA, IIB/TNM (CC)	DFS (p(IIA) = 0.592, p(IIB) = 0.573), **non-significant** N(IIA) = 93, N(IIB) = 27, at 5-year follow up	**Contradictory:** High expression of CXCR3 is not an independent marker for poor prognosis of CRC-II	[61]
**SATB1**	Microarray analysis/IHC	FFPE tissue	I, II & III/TNM (CRC)	OS (*p* < 0.001), RFS (*p* < 0.001), *N* = 328, at 5-year follow up	Overexpression of SATB1 is correlated with LNM, and an indicator of poor prognosis	[65]
**TYMS**	IHC/mRNA expression levels	Tissue	II/TNM (CC)	DFS (HR, 6.07; 95% CI, 0.82 to 44.89; *p* = 0.04), *N* = 120, at 5-year follow up	High TYMS levels and CIMP are associated with poor prognosis in CRC-II	[68]
**CA 19-9**	Meta-analysis	Serum	II/TNM (CRC)	RFS/RR (HR = 2.15, *p* = 0.025), *N* = 384, at 10-year follow up	High levels of CA 19-9 and T4 is associated with poor prognosis in CRC-II	[75]
	Meta-analysis	Serum	II/TNM (CRC)	OCR (HR = 1.63 (1.15–2.32), *p* = 0.006), *N* = 2,515, at 11-year follow up	High preoperative level of CA 19-9 is associated with poor prognosis for CRC-II recurrence	[76]
**CA 125**	Meta-analysis	Serum	II/TNM (CRC)	OCR (HR = 1.87 (1.2–2.8), *p* = 0.003), *N* = 2,515, at 11-year follow up	High preoperative level of CA 125 indicates poor prognosis for CRC-II recurrence	[76]
**ELF3**	Microarray data analysis/IHC	Tissue	II/TNM (CRC)	p(OS) = 0.016, *p*(RFS^a^) <0.001, *p*(ORR) < 0.001, *N* = 185, at 5-year follow up	ELF3 expression is associated with recurrence in CRC-II	[79]
**uPAR**	IHC	FFPE/TMA	B, C/ACPS (RC)	OS (HR = 1.9; *p* = 0.014 in tumour centre and HR = 1.5; *p* = 0.031 in the tumour invasive front), at 5-year follow up	High expression of uPAR in epithelial rectal tumour is linked to poor prognosis in stage B	[80]
**KPNA2**	IHC	FFPE/TMA	II/TNM (CRC)	OS (HR 3.174, 95% CI 2.060–4.889, *p* < 0.001), *N* = 118, at 5-year follow up	High expression of KPNA2 is associated with poor prognosis of CRC-II patients	[93]
**FoxP3**^**+**^**/ CD3**	IHC	FFPE	II/TNM (CRC)	DFS (HR 15.16; 95% CI, 3.43–66.9; *p* < 0.001), *N* = 215, at 5-year follow up	Low FoxP3+/ CD3 significantly correlate with lower survival	[95]

*ACPS* The Australian Clinicopathological System, *CC* Colorectal cancer, *CIMP-High* CpG island methylator phenotype, *CMS4* Consensus molecular subtype 4, *OCR* Overall cumulative recurrence, *CSS* Cause-specific survival, *DFS* Disease-free survival, *FFPE* Formalin-fixed paraffin-embedded, *IHC* Immuno-histochemistry, *LNM* Lymph node metastasis, *MUC2* Mucin 2, *ORR* Overall rate of recurrence, *OS* Overall survival, *RC* Rectal cancer, *RFS* Risk-free survival, *RFS*^a^ Relapse-free survival, *TMA* Tissue microarray.

The literature search was performed primarily on NCBI-PubMed and Google Scholar using the key words “prognostic proteins markers in CRC II patient”, “high risk CRC II”, “stage II prognostic markers” and “stage II CRC”. Articles emphasising the differentiation of CRC-II patients based on the risk of tumour recurrence were initially selected. Subsequently, articles that discriminated high- and low-risk CRC-II groups based on non-molecular factors (primarily histopathology) were excluded. To recognise non-protein based studies, articles on genomic and transcriptomic prognostic markers of CRC-II were briefly mentioned (as above) and cited as they could potentially add value to protein markers. Articles investigating protein markers for lymph-node involvement and those not focused on CRC-II patients were excluded. Consequently, only proteins proposed in the literature as discriminatory between high- and low-risk tumour recurrence groups, along with articles presenting conflicting conclusions, were summarised, described in the text, and listed in Table 2.

### Homeobox Transcription factor (CDX2)

Homeobox Transcription factor (CDX2) is one of three proteins encoded by the ParaHox gene cluster, playing a crucial developmental role in the digestive system of vertebrates [42] and having a particular role as a tumour suppressor in the distal colon [43]. Although CDX2 has been suggested as an indicator of a poor outcome for CRC in general, a bioinformatics approach (after meta-analysis of data sourced from 367 patients in the Cancer Diagnosis Program of the National Cancer Institute (NCI-CDP), 1519 patients from the National Surgical Adjuvant Breast and Bowel Project (NSABP), and 321 patients from Tissue Microarray Database (TMAD) from Stanford USA [33] proposed that CDX2 may be a suitable marker for the prognosis of CRC-II. Using a data-driven informatics approach on a Boolean algorithm (BooleanNet) [44] of expression patterns of genes in the database of 2,329 human colon gene-expression array experiments, the search identified 16 candidate genes from which only one gene (CDX2) expressed into a protein and could be studied for IHC, a clinical grade-diagnostic test [45–47].

Another study evaluated *CDX2* expression using the NCBI-GEO discovery data set and StepMiner algorithm to study 216 CRC-II patients. The association of *CDX2* mRNA levels with difference in 5-year DFS was evaluated and found to be significant (*p* = 0.003) for the 15 *CDX2*-negative tumour patients. Similarly, a significant difference in the 5-year DFS was found in a validation cohort of 121 CRC-II patients when corresponding formalin-fixed paraffin-embedded (FFPE) tissues were tested using IHC with 15 CDX2-negative tumour patients. The study concluded that the absence of CDX2 expression could be used to identify high-risk CRC-II patients who should benefit from chemotherapy [33] (Table 2).

Hansen et al. reached a similar conclusion after performing a study on two unbiased population-based cohorts representing all patients operated on for stage II colon cancer in Denmark in 2002-3 [48]. The study involved 1,157 patients in total, with a significant association found between loss of CDX2 and poor 5-year DFS in both test (*n* = 571, *p* = 0.0267) and validation (*n* = 586, *p* = 0.0118) cohorts. Cohorts were categorised according to CDX2 expression into CDX2-positive, CDX2-moderate and CDX2-negative and DFS rate was found to improve with CDX2 expression as follows: 74%, 72%, and 66% in the test cohort and 75%, 65% and 62% in the validation cohort, respectively. The study concluded that CDX2 can be independently used as a prognostic marker as indicated by DFS data (95% confidence interval 1.129-2.108), *p* = 0.0065.

In another complementary study, the prognostic value of CDX2 was studied by Pilati et al. who concluded that CDX2 as an independent prognostic variable was related to the consensus molecular subtype classified based on the comprehensive gene expression levels across CRC stages. this study found a more useful value in the consensus molecular subtype 4 (CMS4) as opposed to CMS3, CMS2, and CMS1 [49] after analysis of 469 CRC patients. The study was validated on 90 CRC-II patients to suggest that CDX2 absence was an independent poor prognostic marker in terms of OS and RFS [OS: *p* = 0.003; RFS: *p* = 0.027]. Although CDX2 has a stronger significant association with the CMS4 group for both RFS and OS [OS: *p* = 0.007; RFS: *p* = 0.004], similar results are observed in the stage II cohort, though not to a statistically significant level.

However, in a contradictory study, high levels of CDX2 were shown to have a prognostic value for CRC-II. Immunohistochemistry analysis on the FFPE tissue of 227 CRC-II patients using DFS rate as a measure of survival found a significant prognostic relevance for high expression of CDX2 (*p* = 0.03) in cases where MUC2 was also expressed, with the absence of SOX2 (*p* = 0.04) [50]. In the same vein, by first analysing gene expression in 1,045 stage I–IV primary CRCs for CDX2 (n = 403) or IHC (*n* = 642) and in relation to 5-year RFS and OS, a study performed by Bruun et al. showed no significant association (*p* = 0.91) between the absence of CDX2 and the RFS rate in a cohort of 422 CRC-II patients of which 49 were characterised as CDX2-negative. The study suggested that the prognostic value of CDX2 should be limited to CRC-IV patients (p = 4.2 × 10^−10^) [51] (Table 2).

### Carcinoembryonic antigen (CEA)

As CEA levels have been shown to show potential as a diagnostic marker in several types of cancers, including CRC, the prognostic value of CEA was assessed in CRC-II albeit being not very specific nor accurate [52]. CEA is involved in several processes in endothelial cells, including adhesion proliferation and migration [53]. Apart from being proposed as an independent prognostic factor for all stages of CRC [54], one study proposed serum CEA as a robust prognostic marker for stage II tumour recurrence from the National Cancer Database (NCBD) data based on 45,449 patients during the years 2004-14 [55]. Only colon stage I and II cancer patients were included in the study. Results suggested that preoperative CEA levels in serum at or above 2.35 ng/mL can predict CRC-II recurrence. The authors also reported higher 3-year survival rate (79.7% *vs*. 64.5%) for patients with lower levels of CEA. Using the same cutoff of postoperative CEA levels in serum, a more recent study reached the same conclusion after investigating 899 CRC-II patients [56]. The 3-year DFS rate was 88.5% in the ≤ 2.35 ng/mL CEA group and 78.7% in the > 2.35 ng/mL CEA group (*p* = 0.006). Chemotherapy improved survival significantly only in the high-risk CRC-II patients (*p* = 0.09 and 0.03 for DFS and OS).

In addition, the value of early postoperative CEA levels in the prognosis of stage II CRC was investigated by Fengi et al. [57] in a study that included 1,081 CRC-II patients between 2007-15. Using the area under the curve (AUC), a significant relationship between early postoperative CEA and prognosis was demonstrated at an early postoperative CEA cutoff value of 3.66 ng/ml (*p* < 0.001). Furthermore, patients with increased early postoperative CEA had a lower OS (53.62% *vs*. 84.16%), DFS (50.03% *vs*. 86.75%), and CSS (61.77% *vs*. 90.30%) than patients with normal early postoperative CEA levels (*p* < 0.001).

### Chemokine receptor 3 (CXCR3)

Chemokine receptor 3 (CXCR3) regulates leukocyte trafficking, integrin activation and chemotactic migration [58]. It was proposed as a potential prognostic marker in CRC-II patients as it is a significant component of the CXCL10/CXCR3 axis of inflammatory mediators thought to be a lymphocyte-associated metastasis mediator in several cancer types. It was also found that the in vivo receptor knockdown of CXCR3 significantly lessened the spread of the tumour to the liver and lungs [59]. CXCR3 was one of the first markers for CRC-II discovered using RNA microarray technologies. In 2016 in a study by Bai et al., CXCR3 IHC was examined in FFPE tissues of 71 CRC-II patients whose tumour recurred, 72 non-recurrent CRC-II patients and 10 healthy samples (from the peritumoral region) [60]. Data suggested that increased CXCR3 levels increase recurrence risk in CRC-II. Specifically, this study showed poor DFS and OS with the expression of CXCR3 (*p* < 0.001 for DFS and OS). A contradictory study examined expression of CXCR3/4 in 25 CRC-I, 93 CRC-IIA and 27 CRC-IIB patients using IHC found no significant correlation between high expression of CXCR3/4 and 5-year DFS or clinicopathological features using IHC in CRC-II (p(IIA) = 0.592, p(IIB) = 0.573) [61].

### The special AT-rich sequence-binding protein-1 (SATB1)

The special AT-rich sequence-binding protein-1 (SATB1) has been shown to be involved in several cellular processes, such as epidermal differentiation [62], cortical development [63] and embryonic stem cell differentiation [64]. SATB1 was selected based on preliminary microarray analysis and found to have a strong association with lymph node metastasis (LNM). In one study [65], FFPE tissues from 328 patients were analysed by SATB1 IHC and OS of patients with LNM was demonstrated to be associated with an increase in SATB1 levels, especially in patients with CRC-I and CRC-II. The protein levels were monitored for 5 years with *p* < 0.001 for OS and p < 0.001 for RFS. The study concluded that SATB1 may be a prognosticator for LNM and recurrence. In addition, the protein seems to be a potential candidate for poor prognosis in other CRC stages [66].

### Thymidylate Synthetase (TYMS)

Thymidylate Synthetase (TYMS) is essential for supplying the four DNA bases in cells. The enzyme is responsible for the catalysis of the conversion of deoxyuridine monophosphate (dUMP) to deoxythymidine monophosphate (dTMP) [67]. The protein was selected as a potential prognostic marker through its key involvement in DNA synthesis, particularly pyrimidine nucleotide synthesis [36]. Dorada et al. using IHC to detect TYMS expression, examined 120 tumours diagnosed with stage II CRC of which 50% had received chemotherapy after surgery [68] and validated the findings using real-time qRT-PCR demonstrating that adjuvant chemotherapy improved OS (*p* = 0.04) of the cohort. This finding could allow TYMS to decide if chemotherapy may be useful, especially when combined with mismatch repair (MMR) status and the presence of the CpG island methylator phenotype (CIMP or CIMP-High). More studies were conducted across all CRC stages showing that elevated levels of TYMS appear to have a prognostic value with significant chemotherapy benefit after surgery [69, 70].

### Carbohydrate antigen 19-9 (CA 19-9) and 125 (CA 125)

Carbohydrate antigen 19-9 (CA 19-9) was first proposed as a potential marker for gastrointestinal adenocarcinomas using a solid-phase radioimmunometric sandwich assay [71]. It is thought to play a role in cell adhesion processes [72]. Its level in serum is usually measured alongside CEA levels to detect adenocarcinomas [73]. Although its sensitivity is low (23%), and its use as a prognosis factor is controversial [74] with regard to CRC-II, nevertheless, in one study, CA 19-9 levels were measured for all patient’s pre-surgery and tested for association in 384 patients who were followed up over 10 years The OS (HR = 2.15, *p* = 0.025) was determined to be worse with high preoperative levels of CA 19-9 [75] suggesting that CA 19-9 can be used as a prognostic marker for such patients. The study showed that CA 19-9 concentrations and T4 tumour size may predict worse long-term outcomes amongst CRC-II patients.

In a larger cohort of CRC-II,515 CRC-II patients, Xiong et al. [76] investigated the association of overall cumulative recurrence rate (OCR) with preoperative CA 19-9 levels. The levels were checked regularly for participants a month before surgery, with a 5 ng/ml cut-off value. These patients were followed up over 11 years. Using a 5 ng/ml cut-off value, preoperative CA 19-9 level >5 ng/ml were found to associate with a worse OS and an OCR rate (HR = 1.63 (1.15–2.32), *p* = 0.006). The study concluded that CA 19-9 preoperative levels can be used to predict recurrence in CRC-II patients. The same study also investigated carbohydrate antigen 125 (CA 125), a glycoprotein from the mucin family also known as MUC16 [77], showing that preoperative CA 125 levels of >35 ng/ml can indicate a higher OCR for CRC-II patients following surgery.

### E74-like E26 transformation-specific transcription factor 3 (ELF3)

E74-like E26 transformation-specific transcription factor 3 (ELF3) is thought to play a regulatory role in the Wnt/β-catenin pathway [78]. The protein was first suggested as a biomarker using DNA microarray data analysis of 168 patients [35] by Takaoka et al., who suggested it as a marker for poor prognosis. They further investigated ELF3 levels in a cohort of 185 CRC-II patients’ tumour FFPE blocks from 2009-14 using immunohistochemistry (IHC). The study demonstrated that high levels of ELF3 are associated with poor OS (*p* = 0.016) and relapse-free survival (*p* < 0.001) [79].

### Urokinase plasminogen activator receptor (uPAR)

Urokinase plasminogen activator receptor (uPAR) has been proposed as a potential prognostic marker and was uncovered rather serendipitously [80]. It is involved in the plasminogen activation proteolytic cascade in hallmarks of cancer [81], which has been implicated in cancer cell invasion and metastasis [82]. uPAR overexpression, on the other hand, is associated with various types of cancers [83], and therefore has been proposed as a potential prognostic marker to delineate the II and III substages of CRC-II in an attempt to resolve the contradictions between earlier antibody-based studies [84, 85]. In one study on rectal-Dukes B and Dukes C patients, Ahn et al. performed IHC to detect uPAR in the tissue of 170 stage B and 179 stage C rectal cancer specimens staged according to the Australian Clinico-Pathological Staging (ACPS) system with a minimum follow up of 5 years. The study concluded that high uPAR^E^ (i.e., epithelial cell uPAR) expression was associated with poor prognosis, especially in stage B (HR = 1.9; *p* = 0.014 in the tumour center and HR = 1.5; *p* = 0.031 in the invasive tumour front). These data strongly suggested uPAR expressed on tumour epithelial cells had a prognostic value for stage B and C rectal cancer. In general, uPAR expression level has been demonstrated to be independently and negatively associated with the CRC patients survival [86, 87].

### Karyopherin α‑2 (KPNA2)

Karyopherin α‑2 (KPNA 2) was selected as a potential prognostic CRC-II marker as its level is associated with various cancer types [88, 89] and has been investigated in CRC [34, 90]. KPNA2 is a member of the karyopherin α family and is believed to have a significant role in nucleo-cytoplasmic transport [91]. It is also thought to have a role in exporting reactive molecules to the cytoplasm [92]. In a cohort of 118 CRC-II patients, KPNA2 levels were investigated by Jeong et al. as it is usually overexpressed in various types of cancer and known to be related to CRC progression [93]. Corresponding FFPE tissues, with clinical data and with 5-year follow-up, were examined by IHC and expression of KPNA2 determined. The study concluded that the OS rate in CRC-II patients with high KPNA2 was lower than those patients with low KPNA2 (HR 3.174, 95% CI 2.060–4.889, *p* < 0.001), proposing that high KPNA2 expression could be a useful marker of RR.

### Forkhead box P3 (FOXP3) protein and cluster of differentiation 3 (CD3+)

It is thought that Forkhead box P3 (FoxP3^+^) is involved in suppressing the induction of tumour-associated antigen (TAAs) which is expressed in regulatory T cells (Treg) [94]. In one study FoxP3+ and CD3 were closely examined by IHC to evaluate their prognostic significance in the respective FFPE CRC-II tissues of 215 patients [95]. The low expression of both proteins was proposed to significantly indicate a poor prognostic value with a *p*-value of 0.02 and 0.06 for the association of FoxP3 and CD3^+^ with the DFS in the validation cohort. The study demonstrated that combining the levels of these proteins with the mismatch instability (MMS) and tumour stage (T) may help improve segregation between patients who may or may not respond to adjuvant chemotherapy.

### Other potential protein candidates

Over the past years, several alternative protein candidates have been proposed to characterise the distinct stages of CRC. Some candidates are recommended for specific stages, while others distinguish between benign and malignant forms of the disease, including E-cadherin, CD-44, EpCAM, β-catenin, maspin, and vimentin [96, 97]. However, a few can potentially serve as markers specifically for CRC-II. One such example is maspin (mammary serine protease inhibitor), identified as a tumour suppressor gene in various cancers, and its expression demonstrated to correlate with the microsatellite status, tumour dedifferentiation grade, and epithelial-mesenchymal transition trend of the tumour buds [98]. One study showed maspin expression in CRC with high tumour budding compared to low tumour budding. It highlighted that the infiltrative characteristic at macroscopic assessment and nuclear maspin in the tumour buds may associated with the incidence of lymph node metastasis [99], underscoring the potential significance of maspin as a stage II-specific marker. Focused investigations into such markers in the context of CRC-II hold promise for enhancing the precision of diagnostic outcomes in CRC-II.

## Discussion

The appropriate management for CRC-II patients after surgery remains controversial, as opposed to other CRC stages. There remains a strong clinical need for additional molecular biomarker/s as current pathohistological detection techniques to date lack sufficient resolution to discern those patients likely to recur. In fact, a meta-analysis of 18 long-term randomized clinical trials including 2.1 million individuals revealed that common cancer screening tests do not effectively extend lifetime apart from sigmoidoscopy in colorectal cancer [100]. One likely reason is that current pathohistological characterisation of CRC-II cannot account for any micro-metastasis that may be occurring. However, despite this, treatment recommendations (by various cancer authorities) are mostly based solely on pathohistological features. This reliance on pathophysiological features severely limits the ability of available recommendations to be sufficiently accurate to provide adequate decision-making information for better management of CRC-II patients following a potentially cured of resection of the primary tumour.

Although numerous molecular markers have been suggested as potential candidates (Table 2), none can be used independently to delineate between high and low-risk CRC-II patients. Indeed, to date none of them have been unilaterally translated in clinical settings. This may be attributed to several factors that mask the efficiency of current protocols and guidelines in CRC-II management. Firstly, given that the staging systems described by respective cancer bodies differ in drawing the baseline of the histology that discriminates the subdivision of CRC-II stages (A, B and C), the results of independent laboratories will likely reach different conclusions regarding the efficacy of a particular biomarker. Secondly, the selection methodology for biomarker candidates can also play a crucial role in determining the reliability of any selected candidate. Almost all studies used a different selection methodology, some more reliable than others, each reaching different conclusions as to the most effective marker. The technology of choice employed to verify selected candidates may impact the study’s outcome. For example, Den Uil et al. argued that contradictory studies on CDX2 may have arisen from using IHC to determine CDX2 levels in CRC-II patients’ samples instead of mass spectrometry (MS) [101]. When MS was used to analyse samples, a more significant separation was seen (*n* = 31, *p* = 0.011) than using IHC data (*n* = 203, *p* = 0.151).

Thirdly, similar studies may arrive at different conclusions because of the size of the validation or discovery cohorts. The variability in cohort sizes in studies thus far suggests that larger studies may be more reliable than smaller ones, and those uncovering the same markers may be yet more reliable. However, a possible source of contradiction between studies is antibody epitope variation attributed to the different specificity of antibodies developed by different companies can have towards any single potential biomarker. This inherent variability is near impossible to control.

Different protein biomarkers have been proposed to hold prognostic value for better management of CRC-II. Each of these protein prognostic biomarkers has a different efficacy. However, combining the validated biomarkers may open possibilities for better overall prognosis in CRC-II patients, which has yet to be reported in the literature. This method of looking at all possible markers can be performed using rPSL-SWATH MS technology [102] or other such technologies, e.g. multiplex fluorescent antibody IHC that could maximise the chance of identifying the multiple low abundance cancer-associated proteins.

Given the heterogeneity of CRC-II, and for most cancer types, there is a need to individualise treatment and management of the disease through the discovery of accurate and specific prognostic biomarkers. In other words, a shift from reliance on histopathological features and staging to deciding the treatment is required so that better lines are drawn in distinguishing between CRC-II patients in need of chemotherapy after resection and patients cured by surgery alone. The ease with which prognostic protein biomarkers can be integrated cost-effectively into pathology labs means that additional research needs to be directed to fill this gap. Therefore, we suggest that future studies aim to create a panel of protein prognosticators, utilising the available technologies such as rPSL-SWATH MS and IHC to account for low-abundance proteins to reducing morbidity and mortality and improving outcomes for CRC patients worldwide.

The primary benefit of an accurate prognostic assay would be the stratification of high-risk from low-risk stage II patients. This would ensure patients undergo appropriate clinical management, potentially reducing the risk of relapse and improve survival at an estimated $47k AUD per patient (pp) from medical budgets [103]. An accurate prognostic assay would enable the identification of low-risk stage II patients for whom surgery is curative and who do not benefit from additional adjuvant or targeted therapy and also avoid unnecessary psychological burden. These patients are likely to avoid the burden of adverse events from ill-advised intervention. Furthermore, the cost of treating stage II (~$20–32k AUD pp) could be reduced to ~$17k AUD pp by avoiding unnecessary overtreatment [103]. This would not only change the landscape of management of all stage II CRC patients but could potentially improve high-risk patient survival and also benefit low-risk patient overall psychological and physical well-being.

## Supplementary information

Supplementary information

Supplementary Table 1
