# Supplementary material for: Protein prognostic biomarkers in stage II colorectal cancer: implications for post-operative management
Source: BJC Rep. 2024 Feb 13;2:13. doi: 10.1038/s44276-024-00043-z (PMC11523985; doi:10.1038/s44276-024-00043-z)
Supplement: Supplementary file 1 — Supplementary information [file 44276_2024_43_MOESM1_ESM.docx]

# **Stage II staging systems**

To begin to manage and triage patients to prioritise treatment, cancer staging systems have been developed and used for many decades. Cuthbert E. Dukes described the Dukes' cancer staging system in 1932 [1], while the tumour-node-metastasis (TNM) classification was developed by French surgeon Pierre Denoix between 1943 to 1952 [2]. Subsequently, multiple staging systems to classify tumours in CRC (and other cancers) have evolved and continue to be refined (Supplementary Table 1). Clinicians routinely use the pathology-based staging system and histology reports to inform treatment and management plans. This is because in CRC, resection of the tumour has proven to be a highly effective treatment, however, recurrence and postoperative treatment are determined primarily by tumour stage. The staging system, for the most part, has been highly effective and valuable in all CRC stages except stage II, where discrepancies occur.

Various organisations have developed four commonly used systems in CRC staging and have evolved over the past few decades as larger cohorts of patients are studied. They include the AJCC-TNM system [3], which is the most commonly used system [4], the Japanese Classification of Colorectal, Appendiceal, and Anal Carcinoma System (JCCAA) [5], the Australian Clinicopathological System (ACPS) [6] and DUKE [1, 7], a less used staging system (Supplementary Table 1).

The TNM system is the most widely used, mainly based on the histopathological features of the tumour. This system covers most of the features used in other staging systems (Table 1), and it considers the extent of the tumour growth (**T**) if the tumour has reached the lymph nodes (**N**) and the degree of tumour spread (metastasis) to distant organs (**M**). Notably, the ACPS and the TNM systems are both developments of the DUKE staging system with some differences in stage II classification. These differences involve the free serosal surface that defines subcategory B2 in the ACPS system, implying that the tumour is still within reach of any other structure [8]. Once the tumour adheres to other structures, it would only be classified under substage B1 if there is no sign of involvement of the free serosal surface. The ACPS also considered the spread of the tumour and micro-stasis through the lymph node but also the serosa [8].

A few studies suggest that pathological tumour sub-staging can be a useful marker in assessing the tumour's risk of recurrence (RR). One study showed that pathological T4 tumours (pT4) had a lower survival rate than pT3 tumours in CRC-II patients [9]. In another study, researchers suggest CRC-IIA patients (with pT3) may benefit from adjuvant chemotherapy with a 5-year overall survival (OS) of 84.7% as opposed to 72.2% for CRC-IIB patients (pT4a) [10].

# References

1. Dukes, C.E., *The classification of cancer of the rectum.* The Journal of Pathology and Bacteriology, 1932. **35**(3): p. 323-332.

2. Mirsadraee, S., et al., *The 7th lung cancer TNM classification and staging system: Review of the changes and implications.* World J Radiol, 2012. **4**(4): p. 128-34.

3. Weiser, M.R., *AJCC 8th Edition: Colorectal Cancer.* Annals of Surgical Oncology, 2018. **25**(6): p. 1454-1455.

4. Keung, E.Z. and J.E. Gershenwald, *The eighth edition American Joint Committee on Cancer (AJCC) melanoma staging system: implications for melanoma treatment and care.* Expert Rev Anticancer Ther, 2018. **18**(8): p. 775-784.

5. *Japanese Classification of Colorectal, Appendiceal, and Anal Carcinoma: the 3d English Edition [Secondary Publication].* J Anus Rectum Colon, 2019. **3**(4): p. 175-195.

6. Chapuis, P.H., et al., *Staging of colorectal cancer.* Int J Colorectal Dis, 1987. **2**(3): p. 123-38.

7. Dukes, C.E. and H.J. Bussey, *The spread of rectal cancer and its effect on prognosis.* Br J Cancer, 1958. **12**(3): p. 309-20.

8. Charles Chan, P.C. *Notable differences between available clinicopathological staging systems*. Cancer Council Australia 2017 7 November 2017 [cited 2022 28/July]; Guidelines:Colorectal cancer/Differences clinicopathological staging systems]. Available from: <https://wiki.cancer.org.au/australia/Guidelines:Colorectal_cancer/Differences_clinicopathological_staging_systems>.

9. Quah, H.M., et al., *Identification of patients with high-risk stage II colon cancer for adjuvant therapy.* Dis Colon Rectum, 2008. **51**(5): p. 503-7.

10. O'Connell, J.B., M.A. Maggard, and C.Y. Ko, *Colon cancer survival rates with the new American Joint Committee on Cancer sixth edition staging.* J Natl Cancer Inst, 2004. **96**(19): p. 1420-5.
