# Supplementary material for: Protein prognostic biomarkers in stage II colorectal cancer: implications for post-operative management
Source: BJC Rep. 2024 Feb 13;2:13. doi: 10.1038/s44276-024-00043-z (PMC11523985; doi:10.1038/s44276-024-00043-z)
Supplement: Supplementary file 2 — Supplementary Table 1 [file 44276_2024_43_MOESM2_ESM.docx]

**Supplementary Table 1: Description of CRC-II classifications according to major cancer organisations.**

| **Classification System** | **Substage** | **Features** | **Description** |
| --- | --- | --- | --- |
| **The American Joint Committee on Cancer (AJCC) TNM system** | IIA | T3 | - Tumour reached the outermost layers of the colon or rectum but has not passed them (T3), - Not yet grown into adjacent organs, - Not spread to adjacent lymph nodes (N0) or to remote sites (M0). |
|  |  | N0 |  |
|  |  | M0 |  |
|  | IIB | T4a | - Tumour reached the wall of the colon or rectum, - Not passed to other close by tissues or organs (T4a), - Not yet spread to adjacent lymph nodes (N0) or to remote sites (M0). |
|  |  | N0 |  |
|  |  | M0 |  |
|  | IIC | T4b | - Tumour reached the wall of the colon or rectum, - Grown into other neighbouring tissues or organs (T4b), - Not yet spread to adjacent lymph nodes (N0) or to remote sites (M0). |
|  |  | N0 |  |
|  |  | M0 |  |
| **Japanese Classification of Colorectal, Appendiceal, and Anal Carcinoma (JCCRC)** | Stage IIa | T3 N0 M0 | - T3: Tumour invaded beyond the conjoined longitudinal muscle, - N0: No evidence of lymph node metastasis, - M0: No distant metastasis. |
|  | Stage IIb | T4a N0 M0 | - T4a: Tumour invades or perforates the serosa (SE), - N0: No evidence of lymph node metastasis, - M0: No distant metastasis. |
|  | Stage IIc | T4b N0 M0 | - T4b: Tumour directly invades adjacent organs or structures (SI/AI), - N0: No evidence of lymph node metastasis, - M0: No distant metastasis. |
| **Australian Clinicopathological System (ACPS)** | B | B1 | - Tumour reached the muscle layer (muscularis propria), - Not invaded mesothelial surface, - Not spread to lymph node nor to adjacent sites. |
|  |  | B2 | - As B1, however the tumour penetrating the mesothelial surface. |
| **Duke** | B | NA | - The cancer reached the muscle layer of the bowel. - Duke system did not include distant metastases, nor take into account the level of nodal involvement nor the grade of the tumour. |

**T**: Tumour size, **N**: Lymph-node involvement, **M**: Metastasis involvement.
